# Cognition and influencing factors of secondary prevention in patients with ischemic stroke 1 year after discharge in Southwest China: a cross-sectional survey

**DOI:** 10.3389/fneur.2024.1488180

**Published:** 2025-01-07

**Authors:** Xuemin Zhong, Li Li, Qing Ye, Jian Wang, Lanying He, Changqing Li

**Affiliations:** ^1^Second Affiliated Hospital of Chongqing Medical University, Chongqing, China; ^2^Chengdu Second People's Hospital, Chengdu, China; ^3^Affiliated Hospital of Southwest Jiaotong University & The Third People’s Hospital of Chengdu, Chengdu, China

**Keywords:** ischemic stroke, recurrence rate, secondary prevention, patient compliance, lifestyle

## Abstract

Although the risk of recurrent stroke is very high in patients with ischemic stroke (IS), the implementation of secondary prevention of IS has not received enough attention. Therefore, we aimed to investigate the cognition and compliance status of secondary prevention in patients with IS in southwest China and explore the factors affecting compliance with secondary prevention 1 year after discharge. We conducted a cross-sectional survey of patients with IS 1 year after discharge in southwest China through convenience sampling. Factors affecting the compliance of secondary prevention in patients with IS after discharge were analysed. A total of 1,041 patients were included in our study. Nearly one-third of patients did not follow secondary prevention measures according to the guidelines, and an improvement in lifestyle was even less likely. Living with or without children did not significantly affect patient compliance (odds ratio 1.11; 95% confidence interval 0.83–1.49; *p* = 0.47). Furthermore, no significant differences were observed in the probability of treatment acceptance between patients experiencing one or two of the following conditions: hypertension, diabetes, and hyperlipidemia, and those with all three conditions. Thus, patients with IS have insufficient compliance with secondary prevention and there is a particular lack of emphasis on lifestyle improvement. Further interventions are needed to improve compliance with secondary prevention in patients with IS, especially patients with all three conditions of hypertension, diabetes, and hyperlipidemia.

## 1 Introduction

Stroke is the second leading cause of death worldwide and the leading cause of death in China, where one-fifth of the world’s population resides (1, 2). The overall stroke recurrence rate for patients at 3, 6, and 12 months and within 5 years after onset is 12.3, 15.5, and 17.7%, > 40%, respectively (3, 4). Ischemic stroke (IS) and transient ischemic attack (TIA) are the most common types of strokes. Approximately 10–17% patients with IS or TIA have a risk of recurrent stroke in the first year after the onset of symptoms (1–4).

Effective secondary prevention measures, including lifestyle improvements and prevention of risk factors, can reduce IS recurrence and mortality (5–8). According to recent studies, it is possible to prevent up to 90% of strokes by addressing and treating 10 modifiable stroke risk factors, half of which are related to making lifestyle modifications (9, 10). A study conducted by China’s third National Stroke Registry (CNSR-III) found that only 34.9% of patients adhered to guideline-based secondary prevention. Patients who followed the guidelines for secondary prevention had a lower rate of stroke recurrence compared to those who did not (11). Moreover, studies from other countries have indicated that more than one-third to one-half of patients fail to follow long-term strategies for secondary prevention (12, 13).

The incidence of IS varies regionally, with different regions within a country having different incidence and recurrence rates of stroke owing to differences in race, geographical location, and living habits (13–16). China’s southwest region, home to a population of 249 million, contributes 13.3% to China’s GDP. Previous epidemiological investigations on stroke have reported the incidence and mortality rates of IS in southwest China to be 154.6/100,000 and 103.8/100,000, respectively (17). However, despite the high prevalence of stroke in this region, there is a dearth of studies focusing on the investigation and intervention of secondary prevention for patients with IS in southwest China. This study aimed to investigate the cognition and compliance status of secondary prevention in patients with IS in southwest China and explore the factors affecting compliance with secondary prevention 1 year after discharge.

## 2 Materials and methods

### 2.1 Study population

This study was approved by the Hospital Ethics Committee of Chengdu Second People’s Hospital (Ethics approval number: 2024352). Informed consent was obtained from all participants.

We selected patients admitted for IS before June 2022 in Grade III and Class A public general hospitals in Chongqing and Chengdu, which have the highest population density and most developed economy in southwest China, using convenience sampling. The patients met the following diagnostic criteria for IS: age > 18 years, hospitalization for the diagnosis of IS based on the diagnostic criteria of the Chinese Guidelines for the Diagnosis and Treatment of Acute ischemic Stroke 2018 and confirmed through head computed tomography (CT) or magnetic resonance imaging (MRI) (13), patients with a high risk of recurrence, complete medical records and contact information, and patients or their families were willing to participate in the study and signed the informed consent form.

Exclusion criteria were patient death during hospitalization; voluntary discharge; incomplete medical records, such as previous medical history and imaging results of stroke; inability to communicate with investigators and family members owing to critical illness; and refusal to participate in the survey.

### 2.2 Survey content

We classified traditional risk factors such as smoking, hypertension, diabetes, hyperlipidemia, and lifestyle as preventable and controllable factors in line with the Chinese Guidelines for the Secondary Prevention of ischemic Stroke and Transient ischemic Attack 2022 (18). The guidelines cover lifestyle recommendations in four main areas: diet and nutrition, physical activity, alcohol consumption, and obesity.

### 2.3 Survey methods

A hospitalization data survey was used to collect demographic information, such as age, sex, marital status, education level, and cardiovascular risk factors (smoking, drinking, hypertension, diabetes, and hyperlipidemia) through the patients’ hospitalization records.

Patient visits and information survey: Based on patient electronic records, patients or family members were contacted via telephone interviews by four experienced neurologists with unified training. We collected the following patient information: (1) general information (marital status, whether they live with their children, and type of medical insurance); (2) control of risk factors: smoking status, awareness of their disease (including hypertension, diabetes, atrial fibrillation or hyperlipidemia history), or adherence to drug treatment (hypoglycemic, antihypertensive, hypolipidemic, antiplatelet, and anticoagulant therapies); (3) lifestyle improvement (variety of dietary types, low-salt diet, increased activity, alcohol abstinence, and weight loss); and (4) regular hospital visits(make regular visit to the neurology outpatient department of the hospital where you were last treated or a convenient nearby hospital). The follow-up period was between June and September 2023.

### 2.4 Statistical analysis

Continuous variables are presented as means and standard deviations, whereas categorical variables are presented as counts and proportions. We divided the participants into two groups based on their compliance with the doctor’s advice for regular follow-up. We used a t-test to detect statistical differences in continuous variables between the two groups. We employed Fisher’s exact test for categorical variables to identify statistical differences between the groups.

Logistic regression was used to determine the factors that influenced patients with IS who did not have regular follow-up after discharge. Adherence to follow-up procedures was represented as a binary variable (1 = Yes, 0 = No). The above model was used to analyze the data for the two dependent variables. *Patient_i_* represents a vector of patient-level independent variables, including sex, age, marital status, type of medical insurance, and whether the patient lives with a child. *β* is a vector of the parameters of interest, and exp. (β) represents the odds ratio (OR).

Subsequently, we included indicators for hypertension, diabetes, and hyperlipidemia, along with their interaction terms in Model 1. This study aimed to evaluate whether patients diagnosed with multiple cardiovascular diseases (all three conditions) exhibit higher treatment compliance rates than those with one or two of these conditions. Patients with all three cardiovascular diseases were included in the reference group. Statistical analyses were performed using R version 4.1.2 (R Foundation, Vienna, Austria).

## 3 Results

### 3.1 Baseline characteristics

A total of 1,041 patients were followed up in our study. The median age of the patients with regular follow-up at 1 year after discharge was 65.73 years, and the proportion of females was 35.7%. The median age of patients with irregular follow-up was 68.01, and the proportion of females was 37.7%. Additional general information is presented in Table 1.

**Table 1:** Descriptive statistics^a^.

Variables	Follow-up		
	No	Yes	*p*-value
Age, mean (SD)	68.01 (11.45)	65.73 (11.71)	0.006
Female, *n* (%)	101 (37.7)	277 (35.7)	0.609
Marital status, *n* (%)			
Unmarried	2 (0.7)	7 (0.9)	
Married	248 (92.5)	699 (90.1)	0.449
Widowed	14 (5.2)	62 (8.0)	
Divorced	4 (1.5)	8 (1.0)	
Insurance type, *n* (%)			
UEBMI	177 (66.0)	394 (50.8)	
URBMI	67 (25.0)	225 (29.0)	< 0.001
NCMS	21 (7.8)	138 (17.8)	
Self-payment	3 (1.1)	19 (2.4)	
Living with children, *n* (%)	129 (48.1)	414 (53.4)	0.161
Hypertension, *n* (%)	173 (64.6)	551 (71.0)	0.058
Diabetes, *n* (%)	112 (41.8)	314 (40.5)	0.757
Hyperlipidemia, *n* (%)	145 (54.1)	506 (65.2)	0.002
Atrial fibrillation, *n* (%)	13 (0.22)	63(0.27)	0.076
*N*	268	776	

NCMS, New Cooperative Medical Scheme; SD, standard deviation; URBMI, Urban Residents Basic Medical Insurance; UEBMI, Urban Employment Basic Medical Insurance.

^aFor continuous variables, we used a t-test to estimate the p-value; for categorical variables, Fisher’s exact test was used.^

### 3.2 Patients’ knowledge of their illness

Overall, 103, 19, and 45 patients diagnosed with diabetes, hypertension, and hyperlipidemia, respectively, were unaware of their illness (Table 2).

**Table 2:** Patients’ knowledge of their illness.

	Patient perception	Diagnosis of medical record	
		No	Yes
Hypertension	No	281	103
	Yes	39	621
Diabetes	No	599	45
	Yes	19	381
Hyperlipidemia	No	348	92
	Yes	45	559

### 3.3 Secondary prevention in patients with IS 1 year after discharge

Only 18.3% of patients with IS combined with obesity lost weight. Whereas 58% patients abstained from alcohol, 55.5% quit smoking, and less than 80% patients adhered to the three high levels of treatment and antithrombotic therapy (Table 3).

**Table 3:** Compliance with risk factor prevention.

Variables	Compliance rate (%)
Alcohol abstinence	177/305 (58.0)
Low-salt diet	774/1,041 (74.4)
Variety of dietary types	692/1,041 (66.5)
Enhanced activity	730/1,041 (70.1)
Weight loss	53/290 (18.3)
Hypoglycemic therapy	326/424 (76.9)
Antihypertensive therapy	566/723 (78.3)
Hypolipidemic therapy	483/650 (74.3)
Antiplatelet therapy	821/1,041 (78.9)
Smoking cessation	213/384 (55.5)
Anticoagulant therapy	63/76 (81.8)

Rates of smoking cessation, alcohol abstinence, and weight loss were calculated by dividing the number of people who quit smoking, abstained from alcohol, and lost weight by the number of those who smoked, drank, and were obese. The adherence rates to treatment of hyperglycemia, hypertension, and hyperlipidemia were obtained by dividing the number of patients with hyperglycemia, hypertension, and hyperlipidemia by the number of those with hyperglycemia, hypertension, and hyperlipidemia.

### 3.4 Analysis of factors influencing regular follow-up in patients with IS 1 year after discharge

The results of the logistic regression with follow-up as the dependent variable are presented in Table 4. The older the patient, the less likely they were to accept treatment (odds ratio [OR] 0.99, 95% confidence interval [CI]: 0.97–0.99). Furthermore, patients under the New Cooperative Medical Scheme (NCMS) were more likely to accept treatment than those with Urban Employment Basic Medical Insurance (UEBMI) (OR 2.19, 95% CI 1.34–3.74). Patients with hypertension (OR 1.51, 95% CI 1.11–2.06), atrial fibrillation (OR 2.17, 95% CI 1.19–4.26) or hyperlipidemia (OR 1.49, 95% CI 1.10–2.01) were more likely to accept treatment.

**Table 4:** Logistic regression results for follow-up variables.

Variables	OR	95% CI	*p*-value
Intercept	5.33	(0.85, 47.13)	0.09
Age	0.99	(0.97, 0.99)	0.03
Sex (ref = male)	0.85	(0.62, 1.15)	0.28
Education level (ref = illiterate)			
Elementary school or below	0.57	(0.36, 0.90)	0.02
Junior high school or above	0.49	(0.30, 0.80)	0.01
Marital status (ref = unmarried)			
Married	1.23	(0.18, 5.54)	0.8
Widowed	2.07	(0.27, 10.72)	0.42
Divorced	0.88	(0.09, 6.56)	0.9
Insurance type (ref = UEBMI)			
URBMI	1.26	(0.88, 1.81)	0.21
NCMS	2.19	(1.34, 3.74)	<0.001
Self-payment	1.75	(0.55, 7.84)	0.39
Living with children (ref = No)	1.11	(0.83, 1.49)	0.47
Hypertension (ref = No)	1.51	(1.11, 2.06)	0.01
Diabetes (ref = No)	1	(0.75, 1.34)	0.99
Hyperlipidemia (ref = No)	1.49	(1.1, 2.01)	0.01
Atrial fibrillation (ref = No)	2.17	(1.19, 4.26)	0.02

OR, odds ratio; CI, confidence interval; NCMS, New Cooperative Medical Scheme; URBMI, Urban Residents’ Basic Medical Insurance; UEBMI, Urban Employment Basic Medical Insurance.

### 3.5 Correlation analysis of secondary prevention in patients with IS complicated with hypertension, diabetes, and hyperlipidemia

**Figure 1:**
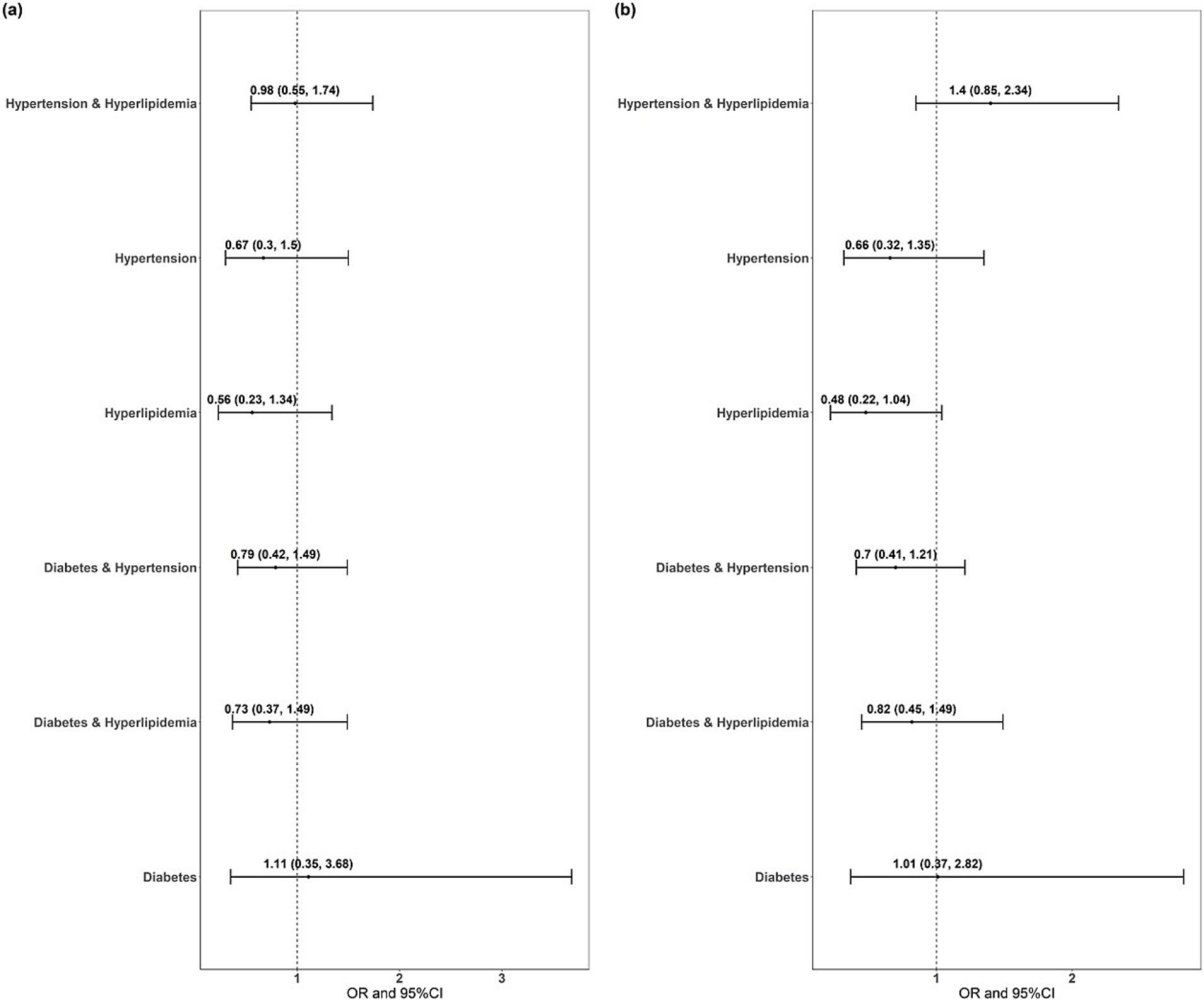
Correlation analysis of secondary prevention in patients with ischemic stroke complicated with hypertension, diabetes, and hyperlipidemia. OR, odds ratio; CI, confidence interval.

Figure 1 displays the ORs between patients with one or two of the three cardiovascular diseases (hypertension, diabetes, and hyperlipidemia) and those with all three. No significant differences were observed in the probability of treatment acceptance between patients experiencing one or two of the three cardiovascular diseases and those with all three, with treatment as the dependent variable (Figure 1A). No significant differences were noted in the probability of treatment acceptance between patients experiencing one or two of the three cardiovascular diseases and those with all three (Figure 1B), with follow-up as the dependent variable. The results align with those of Figure 1A.

## 4 Discussion

According to the investigation of several of Grade III and A general hospitals in Chengdu and Chongqing, we found that the awareness and adherence to secondary prevention measures among patients with IS are not high. Additionally, whether or not patients have children did not significantly impact their compliance. Furthermore, no difference was observed in the prevalence of risk factors between patients with one or two of the three cardiovascular diseases (hypertension, diabetes, and hyperlipidemia) and those with all three.

Although these patients had already experienced an IS, their adherence to secondary prevention was low. A previous study from the Chinese National Stroke Registry-II (CNSR-II) found that the compliance rates with antiplatelet, hypoglycemic, and antihypertensive drugs were 57.58, 63.68, and 61.90%, respectively, 1 year after discharge (1, 7). The results of the Adherence eValuation After ischemic stroke–Longitudinal (AVAIL) study showed that the regimen persistence for secondary prevention medications at 12 months was 65.6% (19). The patients surveyed in our study in 2023 had relatively high medication compliance compared with the CNSR-II study in 2018, which may be owing to the recent health education related to IS. Nonetheless, adherence in western China is lower than that in developed countries. In the AVAIL study, the 12-month persistence was the highest for antihypertensive medications (87.9%), followed by those of antiplatelet (87.1%), diabetes (82.3%), and lipid-lowering (77.6%) medications (19). Other studies, such as Preventing Recurrence of Thromboembolic Events through Coordinated Treatment reported that antithrombotic and statin use was maintained at 98 and 99%, respectively at 1-year follow-up (20). Another study conducted in eastern Canada showed that patients with stroke had a self-reported persistence of 90% for all categories of stroke-prevention medications (21). Our 1-year follow-up results were similar to that of the Riks-Stroke Register (22) in Sweden, which found that persistence by medication category at 2 years post-discharge (56% for statins and 74% for antihypertensive drugs). Taking strong measures for the health education of secondary prevention of AIS is essential.

Age and type of medical insurance affect outpatient follow-up. Similarly, previous studies have found that age and type of medical insurance are factors that affect the medication compliance of patients with IS after discharge (23–25). Our study also found that the proportion of follow-up visits by patients with the NCMS insurance type was higher than that of other insurance types. Chronic disease management in community hospitals has ensured that patients with hypertension, diabetes, and IS are treated with special medical insurance, and the reimbursement ratio is high. However, this may be related to the insurance type of Urban Residents’ Basic Medical Insurance (URBMI) and UEBMI, in which people can visit a pharmacy to buy drugs without registering at a hospital. However, living with children did not promote regular follow-up. This could be attributed to the high work pressure and lack of time among young people to care for patients. Previous studies have shown the significance of caregivers in patient compliance after discharge (26, 27). Therefore, it is crucial to prioritize health education for patients’ families in the future. Furthermore, we observed a lesser focus on lifestyle improvements to prevent recurrent IS compared to regular outpatient follow-up. The cost of improving one’s lifestyle is much lower than healthcare costs. Thus, greater emphasis should be placed on high-value lifestyle interventions, which are consistently reasonable and effective for patients with IS (28–32). Alongside enhancing patients’ medication compliance, it is important to strengthen lifestyle and health education.

Consistent with previous studies, most patients with a history of hypertension and hyperlipidemia in this study had regular follow-up (19, 33). Patients with one or two of the three risk factors had a lower risk of IS recurrence than those with IS having the above three risk factors; however, these patients did not closely monitor their risk factors in our study. Hypertension, hyperglycemia, and dyslipidemia have an evident tendency to aggregate and usually occur in pairs or triplets in the same patient, forming a “two-high” or “three-high” coexistence (34–36). Patients with “three-high” coexistence have an exponentially increased risk of IS recurrence, however, the implementation of “three-high” co-management can produce good health and economic benefits (37, 38). Strengthening secondary prevention interventions for patients with IS at three high levels is essential.

Our findings were based on a real-world study of the cognition and compliance status of secondary prevention measures in patients with IS in southwest China. However, it is important to note that our study had certain limitations. First, we could not analyze the education level of the patients. Second, we did not categorize the type of ischemic stroke in the patients. Third, data collection needs to be more comprehensive, including indicators such as whether the patient is experiencing a first-time stroke, severity of the IS, and functional prognosis of the patients in future research. Fourth, our analyses primarily focused on correlations rather than causal inference; hence, our findings may be susceptible to confounding bias. Fifth, owing to our large sample size, we can explain the secondary prevention of some patients with IS in China. However, there is indeed a lack of generalizability. In the future, an even larger sample size is needed to better investigate the secondary prevention of ischemic stroke in China.

## 5 Conclusion

The recurrence rate of ischemic stroke is high; however, nearly one-third of patients do not perform secondary prevention per the guidelines, particularly for lifestyle improvement. In the future, strengthening the secondary prevention of health publicity for patients with ischemic stroke and their families is essential.

## Data Availability

The raw data supporting the conclusions of this article will be made available by the authors, without undue reservation.

## References

[1] Wu S Wu B Liu M Chen Z Wang W Anderson CS. Stroke in China: advances and challenges in epidemiology, prevention, and management. Lancet Neurol. (2019) 18:394–405. doi: 10.1016/S1474-4422(18)30500-3, PMID: 30878104

[2] Li Z Jiang Y Li H Xian Y Wang Y. China’s response to the rising stroke burden. BMJ. (2019) 364:l879. doi: 10.1136/bmj.l879, PMID: 30819725 PMC6394375

[3] Du W Zhao X Wang Y Pan Y Liu G Wang A. Gastrointestinal bleeding during acute ischaemic stroke hospitalisation increases the risk of stroke recurrence. Stroke Vasc Neurol. (2020) 5:116–20. doi: 10.1136/svn-2019-000314, PMID: 32606083 PMC7337367

[4] Amarenco P Lavallée PC Monteiro Tavares L Labreuche J Albers GW Abboud H. Five-year risk of stroke after TIA or minor ischemic stroke. N Engl J Med. (2018) 378:2182–90. doi: 10.1056/NEJMoa1802712 29766771

[5] Ford ES Ajani UA Croft JB Critchley JA Labarthe DR Kottke TE. Explaining the decrease in U.S. deaths from coronary disease, 1980–2000. N Engl J Med. (2007) 356:2388–98. doi: 10.1056/NEJMsa053935, PMID: 17554120

[6] Asberg S Henriksson KM Farahmand B Asplund K Norrving B Appelros P. Ischemic stroke and secondary prevention in clinical practice: a cohort study of 14,529 patients in the Swedish stroke register. Stroke. (2010) 41:1338–42. doi: 10.1161/STROKEAHA.110.580209, PMID: 20522818

[7] Yan-Xue C Yue J Li Z-X Pan Y Wang Y Wang Y. Status of drug compliance for secondary prevention of acute ischemic stroke and transient ischemic attack in China. Chin J Stroke. (2018) 13:6.

[8] Zeinhom MG Khalil MFE Kamel IFM Kohail AM Ahmed SR Elbassiouny A. Predictors of the unfavorable outcomes in acute ischemic stroke patients treated with alteplase, a multi-center randomized trial. Sci Rep. (2024) 14:5960. doi: 10.1038/s41598-024-56067-5, PMID: 38472241 PMC10933394

[9] Govori V Budinčević H Morović S Đerke F Demarin V. Updated perspectives on lifestyle interventions as secondary stroke prevention measures: a narrative review. Medicina (Kaunas). (2024) 60:504. doi: 10.3390/medicina60030504, PMID: 38541229 PMC10972452

[10] Bailey RR. Lifestyle modification for secondary stroke prevention. Am J Lifestyle Med. (2018) 12:140–7. doi: 10.1177/1559827616633683, PMID: 30202386 PMC6124986

[11] Pan Y Li Z Li J Jin A Lin J Jing J. Residual risk and its risk factors for ischemic stroke with adherence to guideline-based secondary stroke prevention. J Stroke. (2021) 23:51–60. doi: 10.5853/jos.2020.03391, PMID: 33600702 PMC7900402

[12] Chinese Society of Neurology, cerebrovascular group, Chinese Society of Neurology. Chinese guidelines for secondary prevention of ischemic stroke and transient ischemic attack. Chin J Neurol. (2015) 48:258–73.

[13] Liu L Wang D Wong KSL Wang Y. Stroke and stroke care in China: huge burden, significant workload, and a national priority. Stroke. (2011) 42:3651–4. doi: 10.1161/STROKEAHA.111.635755, PMID: 22052510

[14] Yue J Chen W Yong J Yuesong P Zixiao L. Study on the sociological factors of drug compliance for secondary prevention of acute ischemic stroke and transient ischemic attack. Chin J Clin Health Care. (2019) 22:5.

[15] Jia Q Liu L Wang Y. Risk factors and prevention of stroke in the Chinese population. J Stroke Cerebrovasc Dis. (2011) 20:395–400. doi: 10.1016/j.jstrokecerebrovasdis.2010.02.008 20656505

[16] Chinese Society of Neurology, Cerebrovascular Disease Group, Chinese Society of Neurology Bin P. Chinese guidelines for diagnosis and treatment of acute ischemic stroke 2018. Chin J Neurol. (2018) 51:666–82. doi: 10.3760/cma.j.issn.1006-7876.2018.09.004

[17] Wang W Jiang B Sun H Ru X Sun D Wang L. Prevalence, incidence, and mortality of stroke in China: results from a nationwide population-based survey of 480 687 adults. Circulation. (2017) 135:759–71. doi: 10.1161/CIRCULATIONAHA.116.025250, PMID: 28052979

[18] Chinese Society of Neurology, Cerebrovascular Group, Chinese Society of Neurology. Chinese guidelines for secondary prevention of ischemic stroke and transient ischemic attack 2022. Chin J Neurol. (2022) 55:40.

[19] Bushnell CD Olson DM Zhao X Pan W Zimmer LO Goldstein LB. Secondary preventive medication persistence and adherence 1 year after stroke. Neurology. (2011) 77:1182–90. doi: 10.1212/WNL.0b013e31822f0423, PMID: 21900638 PMC3265047

[20] Ovbiagele B Kidwell CS Selco S Razinia T Saver JL. Treatment adherence rates one year after initiation of a systematic hospital-based stroke prevention program. Cerebrovasc Dis. (2005) 20:280–2. doi: 10.1159/000087711, PMID: 16127271

[21] Lummis HL Sketris IS Gubitz GJ Joffres MR Flowerdew GJ. Medication persistence rates and factors associated with persistence in patients following stroke: a cohort study. BMC Neurol. (2008) 8:25. doi: 10.1186/1471-2377-8-25, PMID: 18616796 PMC2474854

[22] Glader EL Sjölander M Eriksson M Lundberg M. Persistent use of secondary preventive drugs declines rapidly during the first 2 years after stroke. Stroke. (2010) 41:397–401. doi: 10.1161/STROKEAHA.109.566950, PMID: 20075360

[23] Ullberg T Glader EL Zia E Petersson J Eriksson M Norrving B. Associations between ischemic stroke follow-up, socioeconomic status, and adherence to secondary preventive drugs in southern Sweden: observations from the Swedish Stroke Register (Riksstroke). Neuroepidemiology. (2017) 48:32–8. doi: 10.1159/000456618, PMID: 28237982

[24] Esenwa C Gutierrez J. Secondary stroke prevention: challenges and solutions. Vasc Health Risk Manag. (2015) 11:437–50. doi: 10.2147/VHRM.S63791, PMID: 26300647 PMC4536764

[25] Han SW Bushnell CD. Stroke secondary medication persistence and risk for hospital readmission within 90 days after discharge. J Neurol Nen. (2016) 7:87–96. doi: 10.21767/2171-6625.100087, PMID: 29801871

[26] Wei JW Wang J-G Huang Y Liu M Wu Y Wong LKS. Secondary prevention of ischemic stroke in urban China. Stroke. (2010) 41:967–74. doi: 10.1161/STROKEAHA.109.571463, PMID: 20224061

[27] Jamison J Sutton S Mant J De Simoni A. Barriers and facilitators to adherence to secondary stroke prevention medications after stroke: analysis of survivors and caregivers views from an online stroke forum. BMJ Open. (2017) 7:e016814. doi: 10.1136/bmjopen-2017-016814, PMID: 28713074 PMC5541606

[28] Wang Y Feng L Zeng G Zhu H Sun J Gao P. Effects of cuisine-based Chinese heart-healthy diet in lowering blood pressure among adults in China: multicenter, single-blinded, randomized, parallel controlled feeding trial. Circulation. (2022) 146:303–15. doi: 10.1161/CIRCULATIONAHA.122.059045, PMID: 35861850 PMC9311470

[29] Lloyd-Jones DM Allen NB Anderson CAM Black T Brewer LC Foraker RE. Life’s essential 8: updating and enhancing the American Heart Association’s construct of cardiovascular health: a presidential advisory from the American Heart Association. Circulation. (2022) 146:e18–43. doi: 10.1161/CIR.0000000000001078, PMID: 35766027 PMC10503546

[30] Wan EYF Fung CSC Yu EYT Chin WY Fong DYT Chan AKC. Effect of multifactorial treatment targets and relative importance of hemoglobin A1C, blood pressure, and low-density lipoprotein-cholesterol on cardiovascular diseases in Chinese primary care patients with type 2 diabetes mellitus: a population-based retrospective cohort study. J Am Heart Assoc. (2017) 6:e006400. doi: 10.1161/JAHA.117.006400, PMID: 28862945 PMC5586469

[31] China Cholesterol Education Program (CCEP) Working Committee, Atherosclerosis Thrombosis Prevention and Control Subcommittee of Chinese International Exchange and Promotion Association for Medical and Healthcare, Cardiovascular Disease Subcommittee of China Association of Gerontology and Geriatrics, Atherosclerosis Professional Committee of Chinese College of Cardiovascular Physicians. China cholesterol education program (CCEP) expert advice for the management of dyslipidaemias to reduce cardiovascular risk (2019). Zhonghua Nei Ke Za Zhi. (2020) 59:18–22. doi: 10.3760/cma.j.issn.0578-1426.2020.01.003 31887831

[32] Hilkens NA Casolla B Leung TW de FE. Stroke. Lancet. (2024) 403:2820–36. doi: 10.1016/S0140-6736(24)00642-1, PMID: 38759664

[33] Wawruch M Zatko D Wimmer G Jr Luha J Kuzelova L Kukumberg P. Factors influencing non-persistence with antiplatelet medications in elderly patients after ischaemic stroke. Drugs Aging. (2016) 33:365–73. doi: 10.1007/s40266-016-0365-2, PMID: 27022917

[34] Grundy SM. Does a diagnosis of metabolic syndrome have value in clinical practice? Am J Clin Nutr. (2006) 83:1248–51. doi: 10.1093/ajcn/83.6.1248, PMID: 16762931

[35] Chen SC Tseng C-H. Dyslipidemia, kidney disease, and cardiovascular disease in diabetic patients. Rev Diabet Stud. (2013) 10:88–100. doi: 10.1900/RDS.2013.10.88, PMID: 24380085 PMC4063094

[36] Weycker D Nichols GA O’Keeffe-Rosetti M Edelsberg J Khan ZM Kaura S. Risk-factor clustering and cardiovascular disease risk in hypertensive patients. Am J Hypertens. (2007) 20:599–607. doi: 10.1016/j.amjhyper.2006.10.013, PMID: 17531915

[37] Wong ND Zhao Y Patel R Patao C Malik S Bertoni AG. Cardiovascular risk factor targets and cardiovascular disease event risk in diabetes: a pooling project of the atherosclerosis risk in communities study, multi-ethnic study of atherosclerosis, and Jackson Heart Study. Diabetes Care. (2016) 39:668–76. doi: 10.2337/dc15-2439, PMID: 27208374 PMC4839178

[38] Sever PS Dahlöf B Poulter NR Wedel H Beevers G Caulfield M. Prevention of coronary and stroke events with atorvastatin in hypertensive patients who have average or lower-than-average cholesterol concentrations, in the Anglo-Scandinavian cardiac outcomes trial–lipid lowering arm (ASCOT-LLA): a multicentre randomised controlled trial. Lancet. (2003) 361:1149–58. doi: 10.1016/S0140-6736(03)12948-0 12686036

